# Expression, Role, and Regulation of Neutrophil Fcγ Receptors

**DOI:** 10.3389/fimmu.2019.01958

**Published:** 2019-08-27

**Authors:** Yu Wang, Friederike Jönsson

**Affiliations:** ^1^Unit of Antibodies in Therapy and Pathology, Institut Pasteur, UMR 1222 INSERM, Paris, France; ^2^Université Diderot Paris VII, PSL University, Paris, France

**Keywords:** neutrophils, Fcγ receptors, IgG, immune complexes, B cells

## Abstract

Neutrophils are best known for their critical role in host defense, for which they utilize multiple innate immune mechanisms, including microbe-associated pattern recognition, phagocytosis, production of reactive oxygen species, and the release of potent proteases, mediators, antimicrobials, and neutrophil extracellular traps. Beyond their well-established contribution to innate immunity, neutrophils were more recently reported to interact with various other cell types, including cells from the adaptive immune system, thereby enabling neutrophils to tune the overall immune response of the host. Neutrophils express different receptors for IgG antibodies (Fcγ receptors), which facilitate the engulfment of IgG-opsonized microbes and trigger cell activation upon cross-linking of several receptors. Indeed, FcγRs (via IgG antibodies) confer neutrophils with a key feature of the adaptive immunity: an antigen-specific cell response. This review summarizes the expression and function of FcγRs on human neutrophils in health and disease and how they are affected by polymorphisms in the *FCGR* loci. Additionally, we will discuss the role of neutrophils in providing help to marginal zone B cells for the production of antibodies, which in turn may trigger neutrophil effector functions when engaging FcγRs.

## Introduction

Neutrophils are key players of the innate immune response. They are the most abundant leukocytes in the human blood (4.5–11 × 10^3^/mm^3^). Following a circadian rhythm, neutrophils are released from the bone marrow (1–3) and circulate in the blood for 4–6 days (4, 5). If they are not attracted to sites of inflammation, they will express markers of aged neutrophils, and preferentially home to the liver, spleen, or bone marrow, where they undergo apoptosis and are cleared by resident macrophages (6–8). This immunologically silent mechanism allows for maintaining a high number of functional neutrophils in the blood (55–70% of all blood leukocytes in the periphery), while guaranteeing a quick removal of deregulated or altered neutrophils. The tight control of neutrophil homeostasis is critical for the organism as many of their effector functions [i.e., production of reactive oxygen species (ROS), release of neutrophil extracellular traps (NETs), or granules containing potent proteases and lipophosphatases (9)] bare the potential to be deleterious for the host and damage surrounding tissues and organs.

Neutrophils express various receptors that enable them to respond almost instantaneously to diverse inflammatory stimuli and danger signals. Among these, receptors for the constant region of IgG immunoglobulins (FcγRs) stand out. They bestow on neutrophils the capacity to react in an antigen-specific way—hence to acquire a key feature of the adaptive immunity. FcγRs enable neutrophils to interact with and respond to monomeric or aggregated immunoglobulins, antigen–antibody immune complexes, and opsonized (antibody-coated) particles, cells, or surfaces. Humans express six classical FcγRs: FcγRI/CD64, FcγRIIA/CD32A, FcγRIIB/CD32B, FcγRIIC/CD32C, FcγRIIIA/CD16A, and FcγRIIIB/CD16B (Table 1). All these FcγRs bind at least two of the four different human IgG subclasses with association constants (*K*_A_) ranging from 8 × 10^7^ down to 2 × 10^4^ M^−1^ (10).

**Table 1:** Classical FcγRs and their expression on neutrophils.

**Structure**	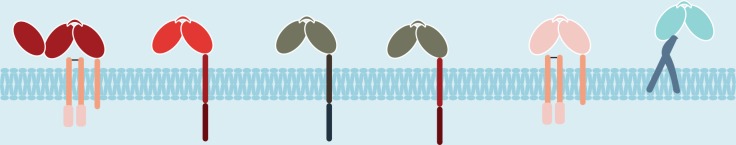					
**Name**	**FcγRI**	**FcγRIIA**	**FcγRIIB**	**FcγRIIC**	**FcγRIIIA**	**FcγRIIIB**
CD	CD64	CD32A	CD32B	CD32C	CD16A	CD16B
Gene	*FCGR1A*	*FCGR2A*	*FCGR2B*	*FCGR2C*	*FCGR3A*	*FCGR3B*
Alleles	–	H_131_ R_131_	I_232_ T_232_	Q_57_ stop_57_	V_176_ F_176_	NA1 NA2 SH
Affinity	High	Low to medium	Low to medium	Low to medium	Low to medium	Low to medium
Expression on resting neutrophils	< 2,000 copies	30,000–60,000 copies	Low to none; increase when 2B4 promotor haplotype	Low to none	Low to none	100,000–200,000 copies
Neutrophil expression in inflammatory conditions	Up to 10-fold increased expression in presence of IFN-γ and G-CSF	Upregulated in presence TNF-α	Low to none; increase when 2B4 promotor haplotype	Low to none	Low to none	100,000–200,000 copies, subject to shedding

All FcγRs, except for FcγRIIB and FcγRIIIB, are classical activating receptors. Their activating signals are transduced by an immunoreceptor tyrosine-based activation motif (ITAM) that is either present in the cytoplasmic domain of the FcγR itself (FcγRIIA/FcγRIIC) or in an associated signaling subunit, notably the FcRγ chain. Upon FcγR aggregation by multimeric ligands, Src family kinases phosphorylate these motifs, allowing the activation of a signaling cascade, involving the spleen tyrosine kinase (SYK), phosphatidylinositol 3-kinase (PI3K), phospholipase C (PLC)-γ, Rho, and Rac, resulting in calcium mobilization, cell activation, cytokine/chemokine production, and cell migration (11–13). Counterbalancing these activating FcγRs, the inhibitory receptor FcγRIIB possesses an immunoreceptor tyrosine-based inhibition motif (ITIM) in its intracytoplasmic domain. Upon its co-engagement with an activating receptor, the phosphorylated ITIM recruits the inositol polyphosphate-5-phosphatase SHIP1 (14) that negatively regulates the signaling cascades initiated by ITAM-containing receptors (15–17). Moreover, several *FCGR* polymorphisms have been described in humans, adding to the complexity of this receptor family with overlapping functions and affinities for their ligands that collaborate, regulate, or compete with each other to tune cellular responses.

In this review, we will focus on IgG receptors (FcγRs) on neutrophils and their role and regulation in steady state and inflammatory conditions.

## Expression and Role of FcγR on Neutrophils During Homeostasis

Blood neutrophils from healthy individuals express large amounts of a rather atypical FcγR, the FcγRIIIB. FcγRIIIB is a glycophosphatidylinositol (GPI)-anchored protein with no signaling capacity on its own. It was first described on neutrophils in 1982 with the means of a newly developed monoclonal antibody (mAb, 3G8) that also recognizes FcγRIIIA on monocytes and NK cells (18). Incubation of neutrophils with 3G8 could efficiently block binding of rabbit IgG-opsonized sheep erythrocytes and soluble rabbit IgG immune complexes (ICs), demonstrating that the newly identified receptor is an IgG Fc receptor (18). FcγRIIIB is one of the most abundant proteins on the surface of neutrophils, with each cell expressing between 100,000 and 200,000 copies (19). In resting neutrophils, the receptor is equally distributed over the cell membrane and is present in both low- and high-density detergent-resistant membranes (DRMs) (20). Additionally, intracellular storage compartments have been described that allow rapid FcγRIIIB mobilization to the cell surface upon receptor engagement (21, 22). Previously thought to have no signaling function, it is now generally accepted that FcγRIIIB can trigger neutrophil activation. Following multivalent cross-linking, FcγRIIIB accumulates in high-density DRMs (20) and elicits downstream signals, leading to Ca^2+^ mobilization, cell adhesion, and degranulation, but not to respiratory burst (23–27). The exact intracellular signaling cascade remains a matter of debate (20, 27–29), but seems to involve phosphorylation of the Src kinase Hck, mitogen-activated kinases (MAPKs) ERK (extracellular signal regulated kinase), and p38 and the tyrosine kinase Pyk2 (30–32). In this context, it is noteworthy that the 3G8 antibody, which is often used to block FcγRIIIB, can trigger intracellular Ca-mobilization and neutrophil aggregation on its own. This cell activation requires co-engagement of another neutrophil FcγR, FcγRIIA *via* the Fc portion of the intact antibody (33). The main functions of neutrophil FcγRIIIB during homeostasis are the removal of spontaneously forming ICs from the vasculature, and the maintenance of the soluble FcγRIIIB (sFcγRIIIB) pool. FcγRIIIB-bound ICs are internalized through a mechanism used by GPI-anchored receptors and fluid-phase endocytosis (27), thereby clearing ICs without triggering further cell activation that could be deleterious to the host. sFcγRIIIB is present in serum of healthy individuals at concentrations of 5 nM (34). It is generated by proteolytic cleavage of surface FcγRIIIB on activated and apoptotic neutrophils (34, 35). Despite its relative abundance, the function of sFcγRIIIB remains elusive. sFcγRIIIB retains Fc-binding capacities and hence competes with membrane low-affinity receptors to dampen Fc-dependent immune reactions (36, 37). Due to the fact that FcγRIIIB binds to multimeric IgG1 and IgG3, but not or poorly IgG2 or IgG4 (10), the biological activity of these latter IgG subclasses should not be affected by sFcγRIIIB. Notably, both IgG2 and IgG4 also bind less well to other low-affinity FcγRs (with the exception of FcγRIIA-H131) (10). Adding to this possible immune-modulatory function, it also has been reported that sFcγRIIIB can bind to complement receptors CD11b/CD18 or CR3/Mac-1 and CD11c/CD18 or CR4 *via* lectin/carbohydrate interactions (38). These interactions can result in cytokine production by neutrophils and monocytes or may inhibit β2 integrin-dependent adhesion and subsequent transendothelial migration (38).

Neutrophils constitutively express a second low-affinity IgG receptor, the FcγRIIA. Albeit less abundant than FcγRIIIB, each neutrophil expresses between 30,000 and 60,000 copies of FcγRIIA (19). Interestingly, this receptor was described to have a lower affinity for IgG on resting than on primed or activated neutrophils (39), a feature that has been attributed to its interactions with integrins (40). Upon efficient cross-linking of FcγRIIA *in vitro*, neutrophils become activated, degranulate, and produce inflammatory mediators and ROS and trigger neutrophil extracellular trap (NET) formation (27, 41–44). More recent data, however, suggest that resting neutrophils rather poorly respond to FcγRIIA-induced activation. One possible explanation for these divergent observations may be found in the purification techniques used to isolate neutrophils. Indeed, density gradient centrifugation or dextran sedimentation used to be the standard techniques. Nowadays, neutrophils are mostly isolated by negative selection procedures that maintain the cells in isotonic buffer, but expose them to magnetic fields. Indeed, while comparative data between the procedures are sparse (45, 46), possible differences in neutrophil priming and purity need to be taken into account when interpreting the data.

In contrast to the abundant FcγRIIIB and FcγRIIA, neutrophils express less than 2,000 copies of the high-affinity FcγRI (19). Ligation of FcγRI on resting neutrophil with a specific antibody does not induce a significant degree of cell activation (47) and neutrophils show poor binding to monomeric IgG1 and phagocytosis of IgG-opsonized particles (48, 49). Neutrophils can also express FcγRIIB; however, its detection is variable among individuals ranging from low to undetectable (27, 50). Although it is well-established that co-engagement of FcγRIIB potently inhibits FcγR-driven cell activation (15–17), it is questionable if the low expression of FcγRIIB on neutrophils could oppose signals generated by the other FcγRs in this context. Finally, a very recent report suggests that neutrophils may also express modest amounts of FcγRIIIA (51), a receptor that was previously thought to be exclusively expressed by NK cells and monocytes (52). In this study, the authors report that FcγRIIIA engagement on neutrophils from FcγRIIIB-deficient and normal individuals efficiently triggers cell activation and mediates phagocytosis of IgG-opsonized beads that could not be blocked by anti-FcγRIIA F(ab′)_2_ fragments (51). Many different groups have studied FcγR expression on neutrophils before, but were incapable to affirm FcγRIIIA expression. This might be due to the fact that most antibodies used to study FcγRIII recognize both FcγRIIIA and FcγRIIIB, and that FcγRIIIB deficiency is rare. In a study from 1994, one can however appreciate some residual FcγRIII [3G8] staining on neutrophils from paroxysmal nocturnal hemoglobinuria patients [a disorder characterized by the deficiency of glycosyl phosphatidylinositol (GPI)-anchored proteins in blood cell membranes] and an FcγRIIIB-deficient patient, as well as on neutrophils treated with GPI-phospholipase C as compared to an isotype control (53). This residual binding, if specific, could support the hypothesis of a low expression of FcγRIIIA on neutrophils. The central piece of evidence for FcγRIIIA expression on neutrophils in the recent report is a single FcγRIIIB-deficient donor, whose genotype was confirmed by RT-PCR and not by sequencing of the FcγR locus. This allows speculation about a cryptic FcγRIIIB expression in this donor. Furthermore, if a low expression of FcγRIIIA can be confirmed in other FcγRIIIB-deficient donors, it will be necessary to clarify to what extent it can contribute to neutrophil effector functions *in vivo*. One point is certain, this study refuels the discussion about the capacity of FcγRIIIB to trigger cell activation and suggests that neutrophil activation observed following receptor cross-linking with anti-FcγRIII F(ab′)2 fragments or ICs can be rather attributed to FcγRIIIA than to the GPI-anchored receptor.

## Neutrophil FcγR in an Inflammatory Context

Neutrophil FcγR expression can change dramatically in the context of an inflammation or infection. Notably, FcγRI is strongly upregulated in the presence of inflammatory cytokines such as interferon-γ (IFN-γ) or granulocyte colony-stimulating factor reaching up to 20,000 copies per cell (19, 48, 54, 55). This confers neutrophils the capacity to efficiently bind monomeric IgG (48), phagocytose IgG-opsonized bacteria (49), exert anti-fungal functions (56), and induce ROS production in response to FcγRI cross-linking (47). FcγRI upregulation also enables neutrophils to efficiently trigger antibody-dependent cytotoxicity (ADCC) (55). As a consequence, neutrophil FcγRI expression has been shown to reflect infection state and disease activity in numerous inflammatory conditions (57–61), and a low CD64 expression is a marker for sustained remission in Crohn's disease patients receiving infliximab (62). Consecutively, neutrophil CD64 has been discussed as an interesting biomarker, especially in the case of sepsis (57). Sepsis diagnosis includes a blood culture that allows specific identification of the disease-causing bacteria and their antibiotic resistances, but takes up to 2 days to generate results. Precious time, during which patients with sepsis suspicion commonly receive broad-spectrum antibiotics to avoid deterioration of their condition. This common practice has it flaws, because antibiotics are not adapted in case of non-bacterial infections, and because some bacteria require specific antibiotics and are not targeted by broad-spectrum ones. Certainly, inappropriate use of antibiotics contributes to the increase of antibiotic resistance among different bacteria that has been classified as “one of the biggest threats to global health, food security, and development today” by the WHO. Neutrophil CD64 expression was proposed as a way to detect infection so that timely decisions and treatment refinement can be made. Neutrophil CD64 can be evaluated within 1–2 h, making it a rapid diagnostic and prognostic marker. Indeed, a study reported not only elevated CD64 expression in septic patients compared to healthy controls, but also a decrease in CD64 expression following treatment with an appropriate antibiotic compared to inefficient treatment (63). However, not all studies find in neutrophil CD64 a reliable marker for sepsis detection, and different studies report divergent sensitivity and specificity for sepsis detection by neutrophil CD64 (64–67). Two meta-analyses report a large heterogeneity in study design and results (68, 69). This may be due to the fact that this test can be run in any laboratory with a flow cytometer and that the results can be expressed either as percentage of neutrophils expressing CD64 or as mean fluorescent intensity of the whole neutrophil population. In the absence of a standardized assay, each laboratory needs to establish its own cutoff. Furthermore, confounding factors (previous use of antibiotics, delayed culture collection, etc.) may result in a negative result from the microbiological test that, as a consequence, poses problems with the classification of the patients. Given the low costs of the assay, neutrophil CD64 remains an interesting candidate to monitor in case of sepsis suspicion. Larger prospective studies are however required to conclude on its sensitivity and suitability as a biomarker, especially in light of new approaches for sepsis diagnosis and the evolution of our understanding of sepsis as a condition involving not only the bacterial infection and its resulting immune response, but also changes in coagulation, immunosuppression, and organ dysfunction (70).

IFN-γ treatment has little effect on neutrophil FcγRIIA expression but can induce FcγRIIB expression (albeit on a low level) and, depending on the experimental conditions, may induce FcγRIIIB down-modulation (19, 71). FcγRIIA expression on neutrophils may however be induced by TNF-α (72). As a consequence, primed neutrophils and neutrophils from individuals with inflammatory conditions show enhanced FcγR-dependent responses (73–75).

Finally, IgG ICs can also be at the onset of inflammation, allergic reactions, and autoimmunity (76, 77). This is notably the case, when the amounts of circulating ICs suddenly rise and exceed the body's capacity to silently remove them, when ICs form that are insoluble and “precipitate” onto endothelial cells, or when autoantibodies bind to large surfaces, i.e., cartilage, thereby opsonizing phagocytosis-resistant structures. All these conditions can be mimicked *in vitro* and helped to understand that soluble ICs require primed neutrophils to efficiently trigger external ROS production and degranulation, while insoluble ICs can activate unprimed neutrophils, leading to intracellular ROS production, degranulation, and sustained liberation of inflammatory mediators such as IL-8 and leukotriene B4 (LTB_4_) that sustain neutrophil-driven inflammation (73, 78). An elegant study, using transgenic mice expressing either FcγRIIA or FcγRIIIB or both in the absence of endogenous activating FcγR, demonstrated that FcγRIIIB has a primordial role in the homeostatic removal of soluble ICs within the vasculature, whereas FcγRIIA engagement by soluble ICs in tissues generates NETs, a pro-inflammatory process linked to autoimmunity (27, 44). Engagement of either FcγR by deposited ICs leads to neutrophil accumulation (44). These data illustrate the specialized role of FcγRs in triggering neutrophil effector functions, despite their overlapping binding properties to IgG.

## Genetic Variations Affecting Neutrophil FcγR Expression and Functions

A number of polymorphisms have been identified in the *FCGR* loci that affect their biological functions and may consequently impact the individual's susceptibility for diseases and their capacity to respond to therapies based on monoclonal antibodies. This is notably the case for polymorphisms that alter the affinity of FcγRs for IgG, thus directly affecting their capacity to clear immune complexes.

Until today, no polymorphism of FcγRI has been identified that modifies the affinity of the receptor for IgG or its associated functions. In contrast, the low-affinity IgG receptor locus on chromosome 1q23.3 coding for all *FCGR2/3* genes is home to multiple genetic variants, including single-nucleotide polymorphisms (SNPs) and copy number variations (CNVs). These genetic variants display heterogeneity among ethnic groups (79). The best-characterized polymorphism of *FCGR2A* results in substitution of an arginine residue by a histidine at position 131 (rs1801274) that results in a receptor variant with an improved binding to IgG2 (and to a lesser extent to IgG1 and IgG3) (80). The *FCGR2A*-*R131* variant is therefore expected to show lower clearance of IgG immune complexes and is indeed associated with susceptibility to auto-immune disorders (81–85) and recurrent bacterial infections with encapsulated bacteria (86). The *FCGR2A*-H131 variant, on the other hand, predisposes individuals to Kawasaki disease and Myasthenia gravis (87, 88). More recently, a splice variant of *FCGR2A*, FcγRIIa(exon6^*^), has been described that retains a cryptic exon in the cytoplasmic tail of the receptor (89, 90). This results in a gain-of-function allele that increases neutrophil sensitivity to IgG stimulation and hence predisposes to anaphylactic reactions following IVIg infusions in patients with hypogammaglobulinemia (71). As described above, FcγRIIB is poorly expressed on neutrophils (50). A specific haplotype in the promoter region, termed 2B.4, was shown to augment FcγRIIB expression on myeloid cells, including neutrophils (91). This promoter variant enables a more efficient binding of the transcription factors GATA4 and Yin-Yang1, resulting in a higher promoter activity and hence higher FcγRIIB expression (92). Surprisingly, this gain-of-function promoter haplotype was found to be associated with systemic lupus erythematous (SLE) (91). A possible explanation might reside in an FcγRIIB-dependent inhibition of IC-phagocytosis, but experimental data to support this hypothesis are still missing. Additionally, a polymorphism of *FCGR2B* (rs1050501) results in the replacement of a threonine by an isoleucine in the transmembrane domain (I232T). The presence of threonine in that position entails a failure of FcγRIIB to enter lipid rafts and, as a consequence, reduces its ability to inhibit activatory receptors (93). For *FCGR3B*, three different allotypes have been described, resulting from five non-synonymous polymorphisms that all affect the neutrophil antigen (NA) located in the membrane-distal Ig-like domain. These variants are termed NA1 (R_36_ N_65_ A_78_ D_82_ V_106_), NA2 (S_36_ S_65_ A_78_ N_82_ I_106_), and SH (S_36_ S_65_ D_78_ N_82_ I_106_) (94). They do not result in detectable differences in affinity for hIgG subclasses (10). The NA1 allotype was nevertheless reported to increase phagocytosis of IgG-opsonized particles (95) and is associated with a reduced responsiveness to IVIG therapy in Kawasaki disease (96). The SH allotype is the rarest allele and less well-characterized. Recently, it has been reported to be associated to increased *FCGR3B* copy numbers (79) that could account for the higher FcγRIIIB expression levels described earlier (97).

A rather large number of studies have associated a single FcγR polymorphism with the induction or severity of antibody-related diseases, or the efficacy of antibody-based therapies. It is however important to bear in mind that all low-affinity IgG receptors are encoded in a single locus on chromosome 1 (1q23). Indeed, a high degree of linkage disequilibrium has been reported for the *FCGR2/3* locus (79, 98, 99) that are strongly linked to ethnic background (79). Association studies should therefore take into account the entire locus and not investigate an isolated gene (100). Adding to the complexity of the 1q23 locus, gene copy number variations (CNVs) have been described for *FCGR3A, FCGR3B*, and *FCGR2C* that directly impact the expression level of the receptors (97). These CNV can include deletions of parts of the locus, giving rise to *FCGR2A/2C* chimeric genes, reducing the expression and function (ROS induction) of the resulting receptor (101). CNVs of *FCGR3B* have been associated to a number of autoimmune disorders, including SLE, rheumatoid arthritis, and systemic auto-immunity (102–106). Indeed, fewer than two copies of *FCGR3B* have been associated to SLE susceptibility (107, 108), which has been confirmed in meta-analysis (109, 110).

Lastly, 0.03–0.1% of the population show a deficiency of *FCGR3B* (and the *FCGR2C* gene) (101, 107). While most studies did not find an association of the *FCGR3B*^null^ genotype with a disease phenotype, one report suggested an association with SLE (102, 111, 112). This apparent contradiction with the finding that low copy numbers increase SLE susceptibility could be due to the low frequency of this genotype in the population, resulting in an insufficient power for calculation. On the other hand, FcγRIIIB-deficient individuals were included at the estimated frequency in a selective cohort of healthy donors with a long list of exclusion criteria, suggesting that FcγRIIIB deficiency remains undetected in most carriers (113). This suggests that *FCGR3B* deficiency does not directly cause disease, but possibly aggravates disease pathogenesis when ICs are accumulating.

## Regulation of Neutrophil FcγRs

The best-described pathway of inhibiting activating FcγRs is, without doubt, through co-engagement of the inhibitory FcγRIIB by the same immune complexes (114, 115). However, as mentioned earlier, human neutrophils express little to no FcγRIIB (50), and it appears therefore mandatory that neutrophils rely on other mechanisms to regulate their activation by FcγRs.

**Figure 1:**
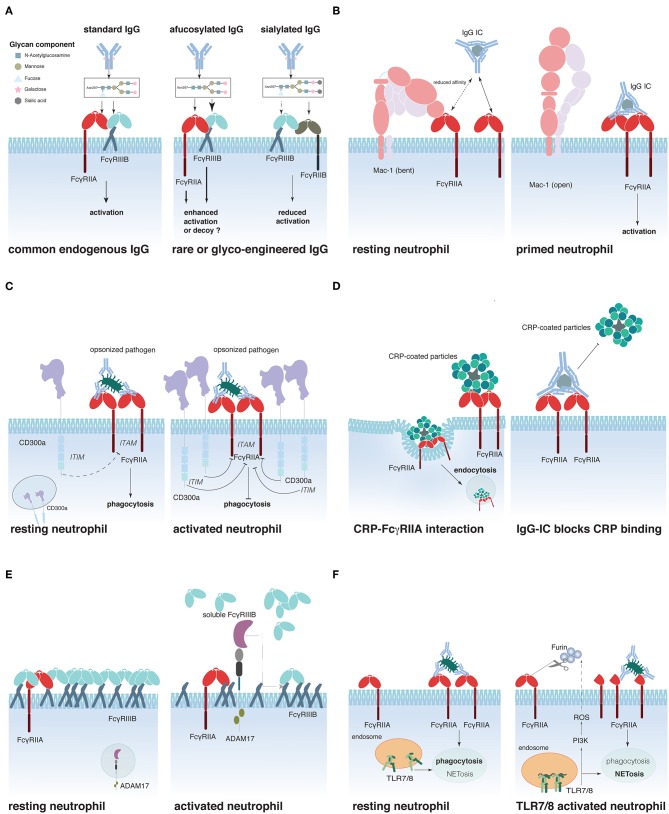
Schematic representation of proposed mechanisms that regulate FcγR activity on neutrophils. **(A)** IgG glycosylation determines its affinity for FcγRs, **(B)** Mac-1 regulates FcγRIIA affinity for IgG, **(C)** CD300a-mediated FcγRIIA inhibition, **(D)** C-reactive protein (CRP)-driven FcγRIIA down-regulation, **(E)** FcγRIIIB shedding by ADAM17, and **(F)** Interplay between Toll-like receptors (TLR)7/8 and FcγRIIA.

Recently, several groups have reported that the glycosylation state of the IgG antibodies significantly modifies their affinity for FcγRs (116–118). Indeed, all human IgG contain a single N-linked glycan positioned at asparagine 297 in the antibody Fc portion, which is critical for their interaction with FcγRs. Several studies have illustrated how the composition of the Fc glycan influences IgG effector functions (119, 120) (Figure 1A). As an example, IgG Fc glycans lacking fucose display a greatly enhanced affinity to the FcγRs, FcγRIIIA, and FcγRIIIB, compared to fucosylated IgG. Besides their well-established improvement of NK cell-dependent ADCC *in vivo* (121–123), afucosylated IgG used to opsonize target cells also activate neutrophils more efficiently than wild-type IgG, inducing pro-inflammatory cytokines and phagocytosis of target cells, but no ROS production or antibody-dependent cellular cytotoxicity activity (124). Addition of an afucosylated anti-CD20 mAb (obinutuzumab) to blood samples from RA and SLE patients resulted in a superior B cell deletion than wt anti-CD20 mAb, concomitantly with neutrophil and NK cell activation (125), suggesting that both cells cooperate to eliminate target cells *in vitro*. Opposing these results, another study reported FcγRIIIB-dependent inhibition of neutrophil ADCC or trogocytosis of solid cancer cells coated with either anti-HER2 mAb (trastuzumab) or anti-EGFR mAb (cetuximab). Notably, copy numbers of FcγRIIIB could be linked to the inhibitory effect and blocking FcγRIIIB with F(ab′)_2_ 3G8 improved target cell killing (126). Further studies are required to determine whether these discrepancies depend on the target cell (solid tumor vs. hematological) and how neutrophil FcγRIIIB (and FcγRIIIA) (51) contribute to cancer elimination *in vivo*. Terminal sialylation of the Fc glycan, instead, decreases the affinity for activating FcγRs (while maintaining the affinity for inhibitory FcγRIIB), and sialylated IgG has reduced capacity to initiate ADCC *in vivo* (118, 127). This might explain why antibodies against autoantigens can often be detected months to years before the first sign of an autoimmune inflammation (128–130). De-sialysation of these antibodies might therefore be a hallmark of autoimmune disease progression (131, 132). In this context, it is noteworthy that a recent report evidenced the potential of *in vivo* glycan engineering of antibodies to modulate of IgG effector functions. Indeed, application of soluble glycosyl-transferases could reduce autoantibody-induced inflammation in models of arthritis and nephrotoxic nephritis (133). However, it remains to be determined whether these very promising findings are exclusively due to IgG glycan modulation or the result of a more complex alteration of multiple glycan structures.

Sialic acid residues have recently been reported to contribute to another mechanism of FcγR regulation (Figure 1B). Prompted by the finding that CD18-deficient neutrophils show an enhanced recruitment to IgG-coated endothelium (134) and that an SNP in the CD18 integrin Mac-1 (rs1143679) is a risk factor for SLE (135), Saggu et al. investigated the interaction between FcγRIIA and Mac-1 on the cell surface. The authors convincingly show that the extracellular portion of Mac-1 in its inactive bend configuration interacts with sialylated FcγRIIA on resting neutrophils and thereby lowers the affinity of the receptor for IgG (40). Interestingly, once the interaction between FcγRIIA and IgG is sufficiently strong to overcome this increased activation threshold, Mac-1 assists FcγRIIA to induce cell spreading (136). This study provides a mechanistic explanation for the observation made earlier that FcγRIIA appears to have a lower affinity for IgG on resting neutrophils than on pre-activated ones.

Other molecules have been suggested to interact and modulate FcγRIIA activity. These include the ITIM-containing CD300a that can be rapidly mobilized from an intracellular pool to the surface of peripheral blood neutrophils following stimulation. Co-engagement of CD300a and FcγRIIA reduced FcγRIIA-dependent activation in an *in vitro* system (137) (Figure 1C). Furthermore, two plasma proteins produced in the liver during the acute phase of inflammations were described to interact with FcγRIIA (and FcγRIIIB), the C-reactive protein (CRP) and the serum amyloid P (SAP) component (138, 139). CRP preferentially interacts with the FcγRIIA-R131-allele and can act like an opsonin, triggering the uptake of CRP-coated particles (140) (Figure 1D). Interestingly, IgG ICs can reduce CRP binding to FcγRIIA, whereas the reverse is not true (141). SAP was similarly described to act as an opsonin that could enhance phagocytosis *via* FcγRs (139). Additionally, it was described to reduce neutrophil adhesion by binding to FcγRIIA (142). Whether SAP binding can regulate IgG-dependent FcγRIIA activation remains however to be determined.

Another possibility to modulate FcγR-induced cell activation is to regulate receptor availability on the cell surface. This can be achieved by receptor internalization (8, 90, 93), translocation from intracellular storage compartments to the cell surface (22), or shedding of the extracellular portion of the FcγR. This latter phenomenon is best documented for FcγRIIIB, which is rapidly and efficiently cleaved from the cell surface following neutrophil stimulation (143), and during neutrophil apoptosis (34, 35). The main protease responsible for this ectodomain shedding appears to be ADAM17 (A Desintegrin and Metalloprotease-17) (144) that is rapidly activated following multiple cell stimulating pathways, such as FcγR/CR-dependent phagocytosis or stimulation with fMLP or PMA (145) (Figure 1E). ADAM17 activation appears to require activation of caspase 8 and mitochondrial ROS production (146). Supporting an important role for the regulation of cell activation by ADAM17, ADAM17 deficiency in humans has been associated with severe inflammatory disorders of the skin and the gut, resulting in recurrent sepsis and poor survival (147, 148). Similar to the ectodomain shedding of FcγRIIIB, reduction of surface FcγRIIA on Langerhans cells and activated neutrophils has been described (39, 149). These initial observations have recently gained attention by the demonstration that TLR-induced activation resulted in the cleavage of extracellular FcγRIIA on the neutrophil surface, thus removing the N-terminal portion of the receptor (150) (Figure 1F). This cleavage has functional consequences for the neutrophils. It reduces their phagocytic activity, while augmenting their propensity to produce NETs, thereby supporting the concept that phagocytosis and NETosis could be neutrophil effector functions that oppose each other, as had been suggested by the finding that NETosis was reduced when microbes where small enough to be phagocytized (151). Similarly, neutrophils from SLE patients and especially their low-density granulocytes that were reported to spontaneously release NETs (152) seem to express less “full-length” FcγRIIA than neutrophils from healthy donors (150). This might explain why neutrophils from SLE patients fail to efficiently clear circulating ICs and are NET-prone (153). The cleavage of the N-terminal portion of FcγRIIA involves a PI3K-dependent production of ROS and seems to be mediated by the serin-protease furin (150); the exact mechanism of its action, however, as well as the fate and role of the N-terminal fragment of FcγRIIA following cleavage remains to be discovered. Similarly, questions on the stability, function, and possibly altered affinity of the shortened membrane-bound FcγRIIA justify further research in this area.

Finally, it has been suggested that FcγRIIIB could represent an efficient modulator of FcγRIIA activity in neutrophils. Indeed, the weakly signaling FcγRIIIB predominantes FcγRIIA expression on resting neutrophils. Furthermore, CD16B extends out further from the cell surface membrane (154, 155), implying that FcγRIIIB is likely to capture circulating immune complexes, thus competing with and preventing FcγRIIA–IgG interactions. The picture is very different with regard to activated neutrophils that, through FcγRIIIB ectodomain shedding, grant access to cell-activating FcγRIIA (154).

## Cross-Talk of Neutrophils and B Cells

Collectively, these studies underline the critical involvement of IgGs in the modulation of neutrophil activity. Indeed, IgGs through FcγRs render neutrophils capable to act to threats to the host in an antigen-specific manner, but are also the trigger for tissue damage if autoantigens are being targeted. Interestingly, there is accumulating evidence that neutrophils are not mere effector cells of the immune system, but actively shape and modulate immune responses through interactions with other cells and the release of mediators. In the context of IgG-dependent immunity, their recently described interactions with B cells are of particular interest.

Data from patients receiving G-CSF suggested that neutrophils can communicate with B cells through production of BAFF (B cell-activating factor) (156), a molecule known to sustain B cell survival and responsiveness (157, 158). Similarly, APRIL (A Proliferation Inducing Ligand) was suggested to be an important survival proliferation factor for human B cells, which additionally drives class-switching reactions (159–161). Neutrophils constitutively secrete APRIL (162), but circulating neutrophils fail to directly activate B cells. Upon infection, neutrophil-derived APRIL is retained by heparan sulfate proteoglycans in mucosal tissues, thereby creating a niche for local plasma cell survival and sustained antibody production (163). Similarly, it has been reported that diffuse large B cell lymphoma secretes chemokines to recruit APRIL-producing blood neutrophils to the tumor (162) and that high APRIL concentrations in tumors are correlated with decreased patient survival rates (164). In SLE, neutrophils interact with B cells in many different ways. SLE neutrophils were reported to show increased expression of BAFF, APRIL, and IFN-α that fuels B cell development and autoantibody production in the bone marrow. In the circulation, SLE neutrophils secrete increased amounts of IL-6 upon IFN-α stimulation that supports survival and maturation of B cells and plasma blasts (165). Concomitantly, they are also NET-prone and release LL37–DNA complexes that trigger polyclonal B cell activation *via* TLR9, giving rise to more NET-specific autoantibodies (166).

Neutrophils can also be found in multiple locations of the spleen, including the perifollicular zone, around the marginal zone (MZ) and the red pulp (167–170). Their exact role in each of these compartments is not fully understood. In the spleen, a specialized subset of neutrophils has been described that has the capacity to provide B cell support. These “B cell helper neutrophils” (N_BH_) are located around the MZ of healthy human donors and express high levels of the B cell-stimulating cytokines BAFF, APRIL, and IL-21 in response to microbial stimuli and thereby provide help to MZ B cells to trigger antibody production against T cell-independent antigens (169). In patients with severe congenital neutropenia (SCN), CD27^+^IgD^low^ circulating MZ B cells and levels of IgM, IgG, and IgA antibodies against T cell-independent antigens were less abundant than in healthy subjects (169). Contradicting this report, no N_BH_ could be identified in spleen samples from organ transplant donors (171); also, similar numbers of CD27+IgD+ memory B cells were reported in patients suffering from chronic idiopathic neutropenia and healthy subjects (172). In addition to BAFF and APRIL, Pentraxin3 (PTX3) has been proposed to be a neutrophil-derived factor that supports B cell functions (173). PTX3 is stored in secondary granules of neutrophils and released upon stimulation with Toll-like receptor agonists (174). PTX3 binds to MZ B cells and enhances the secretion of class-switched IgG in the presence of BAFF (173). The capacity of neutrophils to secrete B cell-stimulating factors enables them to directly interact with the adaptive immune system and shape antibody responses, which, in turn, can trigger potent neutrophil effector functions. This underlines the complexity of our immune system and the multiple layers of regulation that are at play to efficiently protect us from external threats.

## Concluding Remarks

Collectively, the discussed literature exemplifies how our understanding of neutrophils has evolved from their early descriptions as simple first-line defense cells, equipped with powerful weapons to defend the host but unable to differentiate between different threats, to portrayals of cells capable of tailoring their responses according to their environment. We now appreciate that neutrophils interact with various other cell types, integrate complex stimuli, and cooperate with other players of the immune system to fine-tune their responses. The role and regulation of FcγR on neutrophils is not an exception to this rule. Early reports frequently suggested that IgG ICs had very strong neutrophil-activating capacities, but more recently, a much more detailed picture has been drawn, taking into account the size, solubility, composition, and location of ICs. Furthermore, IgG enables neutrophils to function as antigen-specific cells; at the same time, accumulating evidence suggest that neutrophils in turn tune the activity of (at least some) B cells to regulate antibody production. Much remains to be discovered and hopefully new techniques will allow us to unravel previously unappreciated functions of neutrophils and revise others. We are looking forward to hearing more about these exciting cells.

## Author Contributions

All authors listed have made a substantial, direct and intellectual contribution to the work, and approved it for publication.

### Conflict of Interest Statement

The authors declare that the research was conducted in the absence of any commercial or financial relationships that could be construed as a potential conflict of interest.
